# Knowledge, attitude and preventive practices of sexually transmitted infections among unmarried youths in an urban community in Lagos State, Nigeria

**DOI:** 10.4102/phcfm.v12i1.2221

**Published:** 2020-04-21

**Authors:** Esther O. Oluwole, Oluwatobiloba D. Oyekanmi, Doyin O. Ogunyemi, Gbemisola E. Osanyin

**Affiliations:** 1 Department of Community Health and Primary Care, College of Medicine, Faculty of Clinical Sciences, University of Lagos, Lagos, Nigeria; 2 Department of Obstetrics and Gynaecology, College of Medicine, Faculty of Clinical Sciences, University of Lagos, Lagos, Nigeria

**Keywords:** sexually transmitted infections, knowledge, attitude, preventive practices, unmarried youths, community survey

## Abstract

**Background:**

Sexually transmitted infections (STIs) are of public health importance as over 1 million STIs are acquired daily worldwide. One-third of the new cases of curable STIs affect younger persons aged less than 25 years. Sexually transmitted infections can lead to severe complications beyond the immediate impact of infections as such.

**Aim:**

This study assessed knowledge of, attitude towards and preventive practices of STI among young unmarried persons in Surulere local government area (LGA) of Lagos State, Nigeria.

**Setting:**

The study was conducted among young unmarried persons in Surulere LGA, Lagos State, Nigeria, between June and November 2018.

**Methods:**

A descriptive cross-sectional study was conducted among 450 young unmarried persons selected using a multistage sampling technique. An interviewer-administered questionnaire was used to obtain data. Analysis was carried out with Epi-Info 7.2.2.2 software. Chi-square was used to test for associations. Level of significance was at *p* ≤ 0.05.

**Results:**

The mean age was 19.9 + 2.5 years. Majority of the respondents (84.7%) had heard of STIs. About two-third (65.6%) had good knowledge, while majority (98.6%) had good attitude towards the prevention of STIs, but less than half (34.0%) had good preventive practices. Knowledge of STI was statistically significantly associated with age, level of education, attitude and preventive practices of the respondents.

**Conclusion:**

Most of the respondents were aware and had good attitude towards prevention of STI, but gaps exist in knowledge and preventive practices. Hence, targeted education to improve the knowledge and preventive practices against STI among young unmarried persons is recommended.

## Introduction

Worldwide, over a million people acquire a sexually transmitted infection (STI) daily. Every year, there is an estimated 357 million new infections with one of the four STIs globally: chlamydia (131 million), gonorrhoea (78 million), syphilis (5.6 million) or trichomoniasis (143 million).^1^ The United Nations, defines youths as those persons aged between 15 and 24 years.^2^ Young persons (youths) are those in the transition period, from the dependent phase of childhood to the interdependence of adulthood.^3^ Sexually transmitted infections are common among young people, with approximately 20 million new cases of STIs being reported every year in the United States, of which the majority occur among people aged between 15 and 24 years.^4^

Young people are at high risk of getting an STI for the following reasons: young women’s bodies are biologically more prone to sexually transmitted diseases (STDs), lack of access to healthcare, confidentiality concerns and multiple sexual partners, among others.^4^ The Nigerian National Demographic Health Survey (NDHS) in 2008 reported that 16% of young women and 6% of young men aged between 15 and 24 years had sexual debut before the age of 15 years. A survey in Nigeria on the prevalence and predictors of early sexual debut among adolescents reported that an average of 18.6% had sexual debut before their 15th birthday – 16.6% in boys and 20.2% in girls.^5^ This is clearly one of the reasons for the high prevalence of STIs among young people.^6^

Sexually transmitted infections can lead to severe complications beyond the immediate impact of infections. Some STIs, such as herpes and syphilis, can increase the risk of human immunodeficiency virus (HIV) acquisition threefold or more. Gonorrhoea and chlamydia are major causes of pelvic inflammatory disease (PID) and infertility in women.^1^ Data on the incidence and prevalence of STIs in Nigeria are limited because of the under-reporting of STIs, especially among young persons, which is attributable to inadequate diagnostic facilities and limited access to treatment facilities, asymptomatic episodes of the infections and the stigma associated with STI.^7^

Young adults are also exposed to diverse sources of influence (multiple sexual partners, lack of condom use) transecting different levels of causation.^2^ The knowledge of the non-HIV causes of STDs is still lacking, and the risky behaviour practiced by sexually active young adults is on the rise. Prevention of STIs includes counselling, behavioural interventions, comprehensive sexuality education, STI and HIV pre- and post-test counselling, condom promotion and interventions targeted at key populations.^1^ The prevention of STIs, especially in the regions where they are endemic, propelled mainly by heterosexual transmission, includes vaccination (for vaccine-preventable diseases) and practice of the ‘ABC’ approach (abstinence, be faithful to one partner and use of condom).^8^

Literature on the knowledge and preventive practices of STIs in Lagos State is quite scanty, if any at all, especially in Surulere local government area (LGA), which is one of the highly sociable urban areas, with numerous bars, clubs, lounges, malls and pubs, highly populated by young persons. Hence, this study was conducted in Surulere LGA in Lagos State to determine the knowledge of, attitude towards and preventive practices adopted by young unmarried persons against STIs.

## Materials and methods

### Background information to the study area

Lagos State is located in southwestern Nigeria with an estimated population of 21 million as of 2015. Surulere LGA is one of the 20 LGAs in Lagos, a residential and commercial LGA located on the mainland in Lagos State, Nigeria, with a total land mass of 27.1 km^2^. In the last census of the year 2006, there were 503 975 inhabitants, with a population density of 21 864 inhabitants per square kilometre, split across different age groups: 0–14 years, 29.3%; 15–64 years, 67.8%; and 65+ years, 2.9%.^9,10,11^

### Study population, design, sample size determination and selection of participants

The study population consisted of young unmarried youths who must have spent at least 6 months in the selected communities in the LGA. The study was a community-based, descriptive, cross-sectional study. The Cochrane’s formula for descriptive study, *n* = *z*^2^*pq*/*d*^2^, was used, with a standard normal deviation at 95% confidence interval (1.96), a prevalence rate of 62.5% (0.625) from a similar study carried out in Enugu, South East Nigeria,^12^ and an error of precision at 5% (0.05). The minimum sample size was 360. An additional 20% of this (72) was added to allow for possible data loss. Hence, 450 respondents were used for the study. A multi-stage probability sampling technique was used to select the respondents from the study population in five stages. Stage 1 comprised the selection of five wards of the nine wards in Surulere LGA by simple random sampling using the ballot method. In stage 2, 20% of the total number of streets in each of the selected wards was selected through simple random sampling by ballot. Selection of houses was done via systematic sampling, with the sampling interval (*k*) being the total number of houses on the street divided by the number of required houses in stage 3. In stage 4, one household was selected from each of the selected houses, while stage 5 consisted of the selection of one respondent from each household by simple random sampling using the ballot method.

### Study instrument and data collection

A pretested, interviewer-administered, semi-structured questionnaire adapted from the reviewed literature^12,13,14,15,16^ was used for data collection. The questionnaire consisted of four sections: Section A consisted of questions on socio-demographic characteristics of the respondents, while Section B had questions on the knowledge of the respondents about STIs. Section C had Likert-scale questions assessing attitudes of the respondents towards STIs, and Section D comprised questions were asked to determine the preventive practices of the respondents against STIs.

### Data analyses

Data analysis was performed using Epi-info 7.2.2.2. Results were represented in frequencies and percentages. Chi-square test was used to test for associations with the level of significance set at *p* ≤ 0.05.

For the level of knowledge of each respondent about STI, every correct answer attracted one point, while every incorrect answer or non-response attracted a zero. The scores were converted to percentages and graded as poor (< 50%) and good (≥ 50%). For attitude questions on Likert scale, the mean score was calculated. Scores below the mean were classified as poor, while those above the mean were classified as good attitudes. In assessing the preventive practices against STIs, every correct answer scored one point, while an incorrect answer or non-response scored zero. The total score for each respondent was converted to percentage and graded as poor preventive practices if < 50%, and good preventive practices if > 50%.

### Ethical consideration

Ethical approval for this study was obtained from the Health Research and Ethics Committee (HREC) of the Lagos University Teaching Hospital (ADM/DCST/HREC/APP/410). Written informed consent was obtained from each respondent, with assurance of confidentiality of information and their right to withdraw from the study at any point in time. The participants were informed that participation in the study was voluntary.

## Results

### Socio-demographic characteristics of respondents

A total of 425 out of 450 questionnaires administered, which were adequately answered, were analysed with a response rate of 94.4%. The age range and mean age ± standard deviation (s.d.) of the respondents were 15–24 and 19.9 ± 2.5 years, respectively. Most (75.8%) of the respondents were Christians of Yoruba ethnicity (68.0%), and 64.0% had tertiary-level education (Table 1). Majority (84.7%) of the respondents were aware of STI, and most of them (76.7%) got information about STI from the Internet; teachers and schools (76.1%); and electronic media (68.1%).

**TABLE 1:** Socio-demographic characteristics of respondents.

Socio-demographic	Frequency (*n* = 425)	%
**Age (years)**		
15–19	179	42.1
20–24	246	57.9
**Mean age (years)**	19.9 ± 2.5	-
**Sex**		
Male	210	49.4
Female	215	50.6
**Religion**		
Christianity	322	75.8
Islam	99	23.3
Traditional	2	0.5
Others	2	0.5
**Tribe ethnic group**		
Yoruba	289	68.0
Igbo	97	22.8
Hausa	8	1.9
Others	31	7.3
**Level of education**		
No formal education	1	0.2
Primary	11	2.6
Secondary	141	33.1
Tertiary	272	64.0

### Knowledge of respondents about sexually transmitted infections

Viruses (68.3%) and bacteria (48.6%) were the most common known causes of STIs among the respondents. Majority of the respondents (85.8%) knew that HIV or acquired immunodeficiency syndrome (AIDS) is a type of STI; however, a few believed that diarrhoea (23.9%), sickle cell disease (26.7%), tuberculosis (25.4%) and malaria (18.3%) were examples of STIs. Majority (97.8%) of the respondents rightly knew that STIs did not have any gender predilection, as it can affect both men and women, and 96.7% knew that STIs were commonly transmitted by unprotected sexual intercourse. More than half (61.9%) of the respondents also recognised blood transfusion as a means of STI transmission; however, less than half (46.9%) of the respondents identified ‘mother to child’ route as a means of STI transmission (Table 2).

**TABLE 2:** Respondents’ awareness, knowledge of causes, types and transmission of sexually transmitted infections.

Variable	Frequency	%
**Aware of STI (*n* = 425)**		
Yes	360	84.7
No	65	15.3
Source of information (*n* = 360)*		
Electronic media	245	68.1
Print media	180	50.0
Public talks or seminars	210	58.3
Internet	276	76.7
Billboard or posters	124	34.4
Church or mosque	123	34.2
Hospitals or health workers	189	52.5
Teachers or schools	274	76.1
Friends or relations	200	55.6
Causes of STIs*		
Bacteria	175	48.6
Virus	246	68.3
Fungi	157	43.6
Bad hygiene of the man	142	39.4
Bad hygiene of the woman	138	38.3
Drinking unclean water	83	23.1
Having sex during menses	168	46.7
Mosquitoes	66	18.3
Witchcraft	76	21.1
Types of STIs*		
Malaria	66	18.3
Gonorrhoea	255	70.8
Chlamydia	162	45.0
Diarrhoea	86	23.9
Herpes simplex virus	150	41.7
Sickle cell disease	96	26.7
Human papilloma virus	161	44.7
Hepatitis B virus	139	38.6
Syphilis	252	70.0
HIV/AIDS	309	85.8
Tuberculosis	84	25.4
Trichomoniasis	80	22.2
**STIs affect both men and women**		
Yes	352	97.8
No	8	2.2
Transmission of STI*		
Sharing clothes	272	75.6
Sharing needles	197	54.7
Unprotected sexual intercourse	348	96.7
Mother to child	169	46.9
Kissing	123	34.2
Blood transfusion	223	61.9

* , denotes multiple response to the question.

STI, sexually transmitted infections; HIV, human immunodeficiency virus; AIDS, acquired immunodeficiency syndrome.

More than half (58.1%) of the respondents knew that it is possible to be infected with no clinical symptoms. Pain on urination (84.2%) was the commonest STI symptom, while infertility (74.4%) was the most known complication of STI identified by the respondents. In addition, most (64.7%) of the respondents knew that not all STIs can be cured, while majority of the respondents (92.2%) agreed that STIs are preventable. Overall, about three-quarter (66%) of the respondents had good knowledge of STIs (Table 3).

**TABLE 3:** Respondents’ knowledge of symptoms and complications of sexually transmitted infections.

Variable	Frequency	%
**STIs can be asymptomatic**		
Yes	209	58.1
No	151	41.9
Symptoms of STIs*		
Chest pain	271	75.3
Vaginal discharge	297	82.5
Weight loss	105	29.2
Penile discharge	255	70.8
Genital itching	286	79.4
Stomach pain	169	46.9
Pain during urination	303	84.2
Swelling around the genitals	284	78.9
Headache	233	64.7
Blood in urine	232	64.4
Complications of STIs*		
Infertility	268	74.4
Ectopic pregnancy	137	38.1
Miscarriage	208	57.8
Cervical cancer	213	59.2
Testicular cancer	190	52.8
Pelvic inflammatory disease	238	66.1
Premature birth	129	35.8
**All STIs can be cured**		
Yes	127	35.3
No	233	64.7
**All STIs can be prevented**		
Yes	332	92.2
No	28	7.8
**Respondents overall knowledge of STI**		
Good knowledge	236	65.6
Poor knowledge	124	34.4

* , denotes multiple response to the question.

STI, sexually transmitted infections.

### Attitude of respondents towards sexually transmitted infection prevention

Table 4 shows that almost all (95.2%) of the respondents were in favour of educating young people on STI in academic institutions, while the majority (83.3%) agreed that people with STIs should not be isolated. A higher percentage (91.6%) of the respondents agreed with the idea of using condoms during sexual intercourse to prevent STI, while 94.2% believed that screening for STI is good, and majority of the respondents (95.2%) agreed that it was proper to contact the health personnel if one noticed STI symptoms. A large number (74.8%) of respondents agreed that watching pornography can contribute to risky sexual practices that can lead to STI. Overall, majority of the respondents (99%) had a positive attitude towards the prevention of STIs (Table 5).

**TABLE 4:** Respondents’ attitude towards prevention of sexually transmitted infections (*n* = 360).

Statement	SA		A		I		D		SD	
	Frequency	%	Frequency	%	Frequency	%	Frequency	%	Frequency	%
Young people should be educated on STI in academic institutions	300	83.3	43	11.9	16	4.4	0	0	1	0.3
People with STI should not be isolated and avoided	202	56.1	98	27.2	29	8.1	23	6.4	8	2.2
Condoms should be used during sexual intercourse to avoid STI	264	73.3	66	18.3	26	7.2	2	0.6	2	0.6
Screening for STIs is good	262	72.8	77	21.4	17	4.7	3	0.8	1	0.3
If unsure on symptoms of STI, a health personnel should be contacted	269	75.1	72	20.1	13	3.6	4	1.1	0	0.0
Young adults with STI must get treated	280	77.8	69	19.2	8	2.2	3	0.8	0	0.0
I would advise my partner to seek treatment if STI symptoms are noticed	298	83.0	49	13.7	7	2.0	4	1.1	1	0.30
Multiple sexual partners play a role in the transmission of STDs	265	73.6	46	12.8	37	10.3	8	2.2	4	1.1
The use of emergency contraceptive pills does not protect against STI	216	60.0	75	20.8	41	11.4	22	6.1	6	1.7
Watching pornography can contribute to risky sexual practices that can lead to STI	186	51.7	83	23.1	36	10.0	28	7.8	27	7.5

STI, sexually transmitted infections; SA, strongly agree; A, agree; I, indifferent; D, disagree; SD, strongly disagree.

**TABLE 5:** Respondents’ overall attitude towards the prevention of sexually transmitted infections.

Attitude	Frequency (*n* = 360)	%
Positive	355	98.6
Negative	5	1.4

### Respondents’ preventive practices against sexually transmitted infections

Table 6 shows that about a quarter (24.9%) of the respondents were sexually active and more than half (59.4%) of them had sexual debut between the ages of 15 and 19 years. The use of condoms (94.3%), abstinence (86.8%), being faithful to one partner (73.6%) and regular screening (67.9%) were identified as protective measures against STIs. However, less than half (34.9%) of the respondents knew that some STIs can also be prevented by vaccination. Some others (35.9%) were of the opinion that contraceptive use, having sexual intercourse while standing (18.9%), taking drugs before sex (25.5%) and taking alcohol before sex (17.9%) could prevent STIs.

**TABLE 6:** Respondents’ preventive practices against sexually transmitted infections.

Variable	Frequency	%
**Sexually active (*n* = 425)**		
Yes	106	24.9
No	319	75.1
**Age at sexual debut (years) (*n* = 106)**		
10–14	11	10.4
15–19	63	59.4
20–24	32	30.2
Known protective measures against STI among respondents*		
Abstinence	92	86.8
Use of condoms	100	94.3
Faithful to one sexual partner	78	73.6
Regular screening	72	67.9
Contraceptive use	38	35.9
Having sex while standing	20	18.9
Vaccination	37	34.9
Taking drugs before sex	27	25.5
Taking alcohol before sex	19	17.9
**Use of condoms during sexual intercourse among respondent**		
Yes	84	79.3
No	22	20.8
**Frequency of condoms use (*n* = 84)**		
At every intercourse (always)	58	69.0
Often	14	16.7
Rarely	12	14.3
**Number of sexual partners**		
1	57	53.8
2–4	39	36.8
5–8	10	9.4
**Vaccination: Hepatitis B**		
Yes	54	50.9
No	52	49.1
**Vaccination: Human papilloma virus**		
Yes	5	4.7
No	101	95.3
**Overall preventive practice against sexually transmitted infections**		
Good	36	34.0
Poor	70	66.0

* , denotes multiple response to the question.

STIs, sexually transmitted infections.

A large number (79.3%) of the respondents used condoms, and more than half (69.0%) used it always during sexual intercourse. About half (53.8%) of the respondents had one partner, while 49.1% and 95.3% have never been vaccinated for hepatitis B and human papilloma virus, respectively.

Age, level of education, attitude and preventive practices were all found to be statistically significantly associated with the knowledge of respondents about STIs (*p* < 0.05).

## Discussion

Sexually transmitted infections are among the world’s most common diseases, with an annual incidence exceeded only by diarrhoeal diseases, malaria and lower respiratory infections. Each day, almost one million people acquire a new STI; more than 340 million new cases of curable STIs and even more new viral (non-curable) infections occur each year worldwide. Up to 80% of curable STIs occur in developing countries of the world, with adolescents and young adults having the highest rates of these diseases. In developing countries like Nigeria, STIs are among the leading causes of disability adjusted life years (DALYs) lost for women of reproductive age, exceeded only by maternal causes and HIV.^1,2,17,18^

In this study, 450 young unmarried persons were assessed for knowledge of, attitude towards and preventive practices of STI. The mean age of the respondents was 19.9 ± 2.5 years and most (64.0%) had tertiary education, which is in line with similar studies carried out in Shone Town Ethiopia (18.6 ± 1.9) and Malaysia, where majority (78.3%) were undergraduates.^13,15^ This study is however contrary carried out in southwest Nigeria, which reported a lower mean age.^14^ This difference is likely because of age difference in the study population.

Most (66%) of the respondents had overall good knowledge of STIs contrary to a study in Ado-Ekiti, Nigeria, which reported only 6.9% of the respondents as having overall good knowledge of STIs.^14^ This finding could be because of the fact that most of the respondents in this study were undergraduates, compared to the Ado-Ekiti study, which was carried out among secondary school students. Human immunodeficiency virus or AIDS, gonorrhoea and syphilis were the major types of STIs identified by respondents in this study, which is in line with the finding of similar studies conducted within and outside Nigeria.^12,15,19,20,21^

Majority of the respondents in this study knew that unprotected sexual intercourse was a major means of transmission of STIs, while less than half knew about transmission of STIs via mother to child – this finding is consistent with the reports of various other studies conducted within and outside the country.^12,13,14,19^

A statistically significant association was found between age and level of education with knowledge of respondents about STIs (*p* < 0.05), which implies that as the respondents got older or attained a higher level of education, their knowledge also increases. For example, respondents in the age group 20–24 years and those in tertiary institutions had better knowledge of STIs. This finding is similar to that reported by a Malaysian study where students in the age group of 24–30 years were more likely to have good knowledge about STIs, compared to those aged 17–23 years,^15^ which is slightly different from a study conducted in Kwara State, Nigeria, where age was not found to be statistically significant with knowledge, but class do.^22^

With regard to attitude, almost all respondents in this study were of the view that ‘young people should be educated about STI in academic institutions’ – this finding is comparable to that of an Indian study.^23^ Condom use during sexual intercourse to avoid STIs was supported by most of the respondents in this study, which is similar to the finding in the Malaysian study.^15^ The majority of the respondents in this study agreed that multiple sexual partners played a major role in the transmission of STIs,’ while a high percentage of the participants opposed watching pornography. Similarly, other studies in Malaysia, China and Kampala reported that a higher percentage of the respondents knew that having multiple sexual partners was unsafe.^15,24,25^ Overall, almost all respondents in this study had a positive attitude towards prevention of STIs, as was the case with that was conducted in Shone Town, Ethiopia13 This study found a statistically significant connection between knowledge and attitude of the respondents (*p* < 0.05). This implies that as the knowledge of the respondents on STIs increases, they show better attitudes towards its prevention.

**TABLE 7:** Association of age, sex, attitude and preventive practices of respondents with knowledge of sexually transmitted infections.

Socio-demographics	Knowledge grade								
	Poor		Good		Total		Statistical test		
	*n*	*%*	*n*	*%*	*n*	*%*	χ^2^	df	*p*
**Age (in years)**							9.991	1	**0.002**
15–19	65	43.9	83	56.1	148	100			
20–24	59	27.8	153	72.2	212	100			
**Level of education**							17.308†	3	**0.001**
No formal	1	100	0	0	1	100			
Primary	2	28.6	5	71.4	7	100			
Secondary	58	48.3	62	51.7	120	100			
Tertiary	63	27.2	169	72.8	232	100			
**Attitude of respondents**							9.650	1	**0.002**
Positive	119	96.0	5	4.0	124	100			
Negative	236	100.0	0	0.0	236	100			
**Preventive practices of respondents**							7.271	1	**0.007**
Good	30	83.3	6	16.7	36	100			
Poor	40	57.1	30	42.9	70	100			

† denotes Fisher’s exact.

χ^2^, chi-square value; df, degree of freedom; *p*, level of significance value.

About a quarter of the respondents in this study were sexually active during the time of this study; this is in line with the finding in a study in Ogbomoso, Nigeria, and Malaysia.^5,15^ More than half (59.4%) of the respondents in this study had sexual debut between the ages of 15 and 19 years. This finding is similar to that of a study conducted in Ikeji-Arakeji, Osun State, southwestern Nigeria, which reported mean age at first sexual intercourse as 16.8 years (approximately 17 years).^26^ The study in Ogbomoso, Nigeria, found that mean age at sexual debut was 15.80 years in girls and 15.40 years in boys.^5^ Early sexual debut has been reported to be associated with an increased risk of STIs. Researches have shown that the probability of giving birth during the teen years is three times higher for those who had their sexual debut before the age of 16, compared to those who did not.^5^ This report has shown that about 20% of women in Nigeria in 2013 were sexually active by the age of 15 years, and the median age for first sex stood at 17.7 years for women and 20.6 years for men. This declining age of first sexual intercourse has been proffered as one possible explanation for the increase in the number of STIs.^27,28^

Some common misconceptions found among the respondents in this study about the prevention of STIs were: about 36% and 25% felt that contraceptive use and taking drugs before sex, respectively, are known protective measures against STIs, while alcohol intake before sexual intercourse was also regarded as a prevention method by about 18% of the respondents. In a study conducted in China, some of the respondents reported that taking antibiotics before or after sex, taking a shower before or after sex or using a sex detergent wash before or after sex could prevent STIs.^24^

About half of the respondents in this study had one regular partner and the majority used condoms, and most of them used it always (at every sexual intercourse). This finding is contrary to the report of a study among youths in Nairobi where less than half of the respondents use condoms and in southern Ethiopia where 29.0% use condoms and 11.9%, respectively, use it always.^29,30^ These differences probably could be because of differences in the cultures and religious beliefs in the different study settings. The study in Malaysia reported that about two-third of the respondents had only one partner.^15^ These findings point to a higher rate of risky sexual behaviours among young unmarried people in the different study settings. About half (51%) of the respondents in this study have been vaccinated against hepatitis B virus, while almost all (95%) were not protected against human papilloma virus. Pre-exposure vaccination has been recommended as one of the most effective methods for preventing transmission of human papillomavirus (HPV), hepatitis A virus (HAV) and hepatitis B virus (HBV). Human papillomavirus vaccination is recommended routinely for boys and girls aged 11 or 12 years beginning at 9 years of age.^31^

Overall, about two-third (66.0%) of the respondents in this study had poor preventive practices against STIs. Knowledge of respondents of STIs was found to be statistically significant with the preventive practices of STIs (*p* < 0.05).

## Conclusion

Most of the respondents in this study were aware of and had good attitude towards the prevention of STI, but gaps still exist in their knowledge of STIs. Preventive practices of STIs are very poor among the respondents. Therefore, targeted education on behaviour change communication aimed at young unmarried persons in communities is highly recommended to improve their knowledge and thereby implementation of preventive practices against STIs.

## Limitations

This study had some limitations. Firstly, our study participants were from only one LGA out of the 20 in Lagos State. This study was conducted in a community, and the results represent the views of the respondents in that community alone, which might be different from others in other parts of the state. The presence of recall bias in this study is also very likely. Therefore, further research with a larger population with adequate power and sample sizes is recommended.

## References

[1] World Health Organization Sexually transmitted infections (STIs) [homepage on the Internet]. 2016 [cited 2018 May 07] Available from: http://www.who.int/mediacentre/factsheets/fs110/en/

[2] World Health Organization Global strategy for the prevention and control of sexually transmitted infections: 2006–2015: Breaking the chain of transmission. Geneva: World Health Organization; 2006.

[3] United Nations, Educational, Scientific and Cultural Organization (UNESCO). Learning to live together. What do we mean by youth? [homepage on the Internet]. [cited 2018 May 12]. Available from: www.unesco.org/new/en/social-and-human-sciences/themes/youth/youth-definition

[4] Centers of Disease Control and Prevention Sexually transmitted diseases. CDC Fact Sheet: Information for teens and young adults: staying Healthy and Preventing STDs [homepage on the Internet]. [cited 2018 May 14]. Available from: https://www.cdc.gov/std/life-stages-populations/stdfact-teens.htm

[5] Fehintola FO, Fehintola AO, Ogunlaja OA, et al Prevalence and predictors of early sexual debut among adolescents in Ogbomoso, Nigeria. Am J Public Health Res. 2018;6(3):148–154. 10.12691/ajphr-6-3-4

[6] National Population Commission (NPC) Nigeria and ICF Macro Nigeria demographic and health survey 2008. Abuja: National Population Commission and ICF Macro, 2009; p. 225–238.

[7] Omobude-Idiado SN, Bazuaye GN Patterns of sexually transmitted infections (STIs) reported among students in a federal university in Midwestern Nigeria. Coll Stud J. 2009;43(2):384–390.

[8] Shelton JD, Halperin DT, Nantulya V, Potts M, Gayle HD, Holmes KK Partner reduction is crucial for balanced ‘ABC’ approach to HIV prevention [serial online]. BMJ. 2004 [cited 2018 May 14]. Available from: https://www.ncbi.nlm.nih.gov/m/pubmed/15073076/10.1136/bmj.328.7444.891 PMC387490 15073076

[9] Surulere LGA in Lagos State [homepage on the Internet]. [cited 2019 July 05]. Available from: https://www.citypopulation.de/php/nigeria-admin.php?adm2id=NGA025020

[10] Lagos State Government Surulere [homepage on the Internet]. [cited 2018 Jun 03]. Available from: http://www.lagosstate.gov.ng/entities.php?k=109

[11] Surulere Local Government Area in Nigeria. City population [homepage on the Internet]. [cited 2018 Sep 07]. Available from: citypopulation.de

[12] Chindinma N, Ekenechukwu Y, Birinus E, Christian O, Ikenna O HIV and sexually transmitted infections knowledge and practices; a survey of female secondary school students in Enugu, South East Nigeria. J Med Res. 2017;3(2):66–70. 10.31254/jmr.2017.3208

[13] Kejela G, Soboka B Assessment of knowledge, attitude and preventive practice towards sexually transmitted infections among preparatory school students in Shone Town, Ethiopia 2014. J Health Med Inform. 2015;6:183 10.4172/2157-7420.1000183

[14] Amu EO, Adegun PT Awareness and knowledge of sexually transmitted infections among secondary school adolescents in Ado Ekiti, South Western Nigeria. J Sex Trans Dis. 2015;2015:1–7. 10.1155/2015/260126 PMC4546807 26345225

[15] Folasayo AT, Oluwasegun AJ, Samsudin S, Saudi SN, Osman M, Hamat RA Assessing the knowledge level, attitudes, risky behaviors and preventive practices on sexually transmitted diseases among university students as future healthcare providers in the central zone of Malaysia: A cross sectional study. Int J Environ Res Public Health. 2017;14(2):159 10.3390/ijerph14020159 PMC5334713 28208724

[16] Makwe E, Adenyuma MO Awareness of sexually transmitted infections (STIs) including HIV/AIDS among undergraduates’ students of university of Abuja Nigeria. Br J Appl Sci Technol. 2014;4(4):705–717. 10.9734/BJAST/2014/6102

[17] Sexually transmitted infections in developing countries: Current concepts and strategies on improving STI prevention, treatment, and control. In: CDC 2005 STD treatment guidelines [homepage on the Internet]. [cited 2019 July 05]. Available from: http://www.cdc.gov/std/treatment/

[18] World Health Organization Global strategy for the prevention and control of sexually transmitted infections: 2006–2015. Breaking the chain of transmission; who. int. 2007; p. 61.

[19] Ash FK, Kaya S, Husrev D, Evren H Knowledge, attitudes and behavior towards sexually transmitted diseases in Turkish Cypriot. Cent Eur J Public Health. 2013;21(1):54–58. 10.21101/cejph.a3808 23741902

[20] Norbu K, Mukhia S, Tshokey Assessment of knowledge on sexually transmitted infections and sexual risk behavior in two rural districts of Bhutan. BMC Public Health. 2013;13:1142 10.1186/1471-2458-13-1142 25292443 PMC3890549

[21] Santos-Hovener C, Marcus U, Koschollek C, et al Determinants of HIV viral hepatitis and STI prevention needs among African migrants in Germany; a cross-sectional survey on knowledge, attitudes, behaviors and practices. BMC Public Health. 2015;15:753 10.1186/s12889-015-2098-2 26246382 PMC4545823

[22] Oluyemi JA, Yinusa MA, Abdullateef R, Akoh S, Kehinde K Knowledge of sexually transmitted diseases among secondary school adolescents in Asa local government area of Kwara State Nigeria. African Sociological Review. 2015; 19 (1): 64–76.

[23] Nagesh TS, Akhilesh A knowledge and attitude about sexually transmitted infections other than HIV among college students. Indian J Sex Transm Dis AIDS. 2017;38(1):10–14. 10.4103/0253-7184.196888 28442798 PMC5389207

[24] Zhang D, Pan H, Cui B, Law F, Farrar J, Ba-Thein W Sexual behaviors and awareness of sexually transmitted infections among Chinese university students. J Infect Dev Ctries. 2013;7(12):966–974. 10.3855/jidc.3872 24334944

[25] Sekirime WK, Tamale J, Lule JC, Wabwire-Mangen F Knowledge, attitude and practice about sexually transmitted diseases among university students in Kampala. Afr Health Sci. 2001;1(1):16–22. 12789128 PMC2704443

[26] Akokuwebe ME, Daini B, Falayi EO, Oyebade O Knowledge and attitude of sexually transmitted diseases among adolescents in Ikeji-Arakeji, Osun State, in South-Western Nigeria. Afr J Med Med Sci. 2016;45(3):281–289. 29462534

[27] National Population Commission (NPC) [Nigeria] and ICF International Nigeria demographic and health survey 2013. Abuja: NPC and ICF International; 2014.

[28] Adler MW Sexually transmitted infections in Europe. Eurohealth. 2006;12:3–6.

[29] Nega DM, Sindew MA, Befikadu TG, Girum ST, Zeleke AM, Eshetu ZT Knowledge, attitude and preventive practices towards sexually transmitted infection among preparatory school students of Arsi Negelle Town. J AIDS Clin Res. 2017;8(12):748 10.4172/2155-6113.1000748

[30] Wairimu HW Knowledge, attitudes and practices concerning, HIV/AIDS prevention among youth in East Leigh location in Nairobi County Research project submitted for the Award of Masters of Arts in Sociology. University of Nairobi, 2014.

[31] Workowski KA, Gail A, Bolan GA Sexually transmitted diseases treatment guidelines. MMWR Recomm Rep. 2015;64(RR-03):1–137. PMC5885289 26042815

